# On the Congenital and Hereditary Transmissions of Diseases and Pathological Peculiarities, Especially Those of the Human Teeth; an Inaugural Thesis Respectfully Submitted to the Faculty of the Baltimore College of Dental Surgery, for the Degree of Doctor of Dental Surgery, February 20th, 1858

**Published:** 1858-04

**Authors:** J. G. Russell


### ARTICLE IV.

*On the Congenital and Hereditary Transmissions of Diseases and Pathological Peculiarities, especially those of the Human Teeth; an Inaugural Thesis respectfully submitted to the Faculty of the Baltimore College of Dental Surgery, for the Degree of Doctor of Dental Surgery, February 20th,* 1858, *by* J. G. RUSSELL, M. D.

IT is designed in this paper to show not only by analogy, but by a collection of facts, that accidental defects and diseases, as well as natural peculiarities and marks, may be transmitted congenitally; and that where such defects depend on established habits, or long and continued practices, from one generation to another, the offspring are influenced by such habits, both in formation of body, and quality of mind.

The principal object, however, will be to show that in no part of the economy are such defects transmitted with more certainty than in the human teeth.

It has often been said that "man is a creation of habits;" another has observed "we are a bundle of habits." With equal propriety may it be said, that we derive very many qualities, and marked characteristics from the long continued habits or customs of our progenitors. It is difficult to draw a distinct and well defined line, that shall, without exception, constitute an infallible rule. We can almost every day witness some departure from what may be said to constitute a law of nature, or the rule of action by which nature seems to be governed. There are many things in nature that can admit of no previous demonstration, yet we rely with great confidence upon the usual results. For instance, we all readily accede to the proposition of the Scripture, and fully believe that "whatsoever a man soweth, that shall he reap." This declaration was predicated on observation, and in most instances is literally true, though it is not without exception, for the husbandman finds that sometimes after sowing one kind or variety of seed, he will reap another. Nevertheless this is so generally true that it may safely be taken as the rule or law of nature. The wisdom of experience and age teach the husbandman to select his seed of varieties of the best growth, and most perfect development. If he plant corn, he will select every ear not only with regard to size of the kernel, but he is equally careful that it shall be free from decay and unsoundness.

If he grow wheat, his seed must be of large and well developed grains, clean and wholly devoid of every mark of rust, smut, blast or blight. Now why is this? Is it not because experience teaches him that whatsoever he soweth, that shall he reap?

To come one step nearer our subject in a practical sense, let us observe the principles that actuate the raisers or breeders of domestic animals. They have long since learned this grand secret from experience, and are governed by it, as the perfection and beauty of very many of our most useful and valuable animals bear witness. To such perfection and certainty is this principle or capability carried, that we can calculate almost to a certainty as to what will be the qualities, capacity or capability of an animal, before its existence, for speed, for any particular gait or distance, as well as its size, disposition and constitution.

By the observance of certain known laws, we may raise at will a horse particularly suited to the saddle, another to the harness, a third for the dray or heavy draft; while we are enabled to produce, at pleasure, the beautiful, elegant and fleet racer, that can almost untiringly sweep over the course with the velocity of the deer, and with a seeming animation, and love for its task, that challenges our admiration for its sport, rather than our sympathies for its toils and fatigues.

The force of habit, or rather the congenital transmission of physical changes in the offspring, by a custom practiced for years or a long time, upon the progenitors, is a thing not only incredible, to the casual observer, but strange and inexplicable, to the most thoughtful and observing; we are however, forced to submit to positive and well authenticated facts, notwithstanding we may be at fault in theory, and wholly unable to give a clear and satisfactory explanation as to all the mŏdes of operation by which certain known results are produced. For instance, who can satisfactorily explain how or by what law of nature, the following results are produced? In England, it was formerly, for a long time, in certain localities, the custom to crop the ears of all the horses, both male and female, until at length the ears of the colts of these animals were in fashion naturally; and as short and clumped as the most fastidious could desire. This is so strange, and taxes belief so strongly, that I will furnish competent evidence in support of the statement.

I shall refer to the learned and useful work of Professor "Youatt on the Horse," quoting the writer's own words. He says, "The hearing of the horse is remarkably acute. A thousand vibrations of the air, too slight to make any impression on the human ear, are readily perceived by him. It is well known to every hunting-man that the cry of the hounds will be recognized by the horse, and his ears will be erect, and he will be all spirit and impatience a considerable time before the rider is conscious of the least sound. Need any thing more be said to expose the absurdity of cropping. This custom of cutting the ears of horses originated (to its shame) in Great Britain, and for many years was a practice cruel to the animal, depriving him of much of his beauty; and so obstinately pursued, that at length the deformity became in some *hereditary*, and a *breed* of *horses born without ears were produced!*"

I will also quote a few words, in proof, from the excellent work of the celebrated French veterinarian, Prof. Gragnier. Speaking of this abominable custom, he says, "And thus the English completely destroy or disfigure two organs which embellish the head of the most beautiful of all animals, and which, by their various motions, indicate the thoughts that are passing through his mind, the passions which agitate him, and especially the designs which he may be meditating, and which it is often of great importrnce to learn, in order to guard against the danger which may be at hand."

Taking it for granted that my object is quite apparent in making these and like statements, I will refer to the susceptibility of the horse in one instance more, to transmit to its progeny not only its habits, acquired by training, but its physical adaptation to the practicing such habits. It is a known fact, that in all the southern and western slave states, we consider it an indispensable requisite that the saddle-horse shall "rack" or "pace."

Every one acquainted with the habits of the horse originally, knows that this gait is an unnatural one, and wholly the result of much training and practice. But we now find springing from these trained animals, a *breed* of horses that prefer this pace to all other gaits; and, in fact, are swifter in this movement than in any other. Not only this, but we find them conforming to this force of habit in the natural and perfect adaptation of their shape, giving them all the facilities for this peculiar movement, and you will now find this breed of pacers to be high formed on the withers, with a heavy and strongly muscled arm, and sloping from the hip to the round of the thigh, and with a remarkably flat and crooked hind leg. Any one acquainted with the necessary qualifications of a pacing saddle-horse, knows this to be precisely the form and make best adapted to this (originally) unnatural action in the horse.

How strikingly does this prove the proposition with which we set out, and particularly when we show by history, as well as the laws that govern the procreation of distinct species (and the propagation from hybrids) that all the varieties and different breeds of horses, are merely the result of advantages derived from a knowledge of facts as above stated. Perhaps the dog can furnish examples of this principle, quite as familiar to all, as almost any of the domestic animals. The dog is an animal which seems to have been destined by the Creator to be the friend and assistant of man. Throughout the dangers which beset the human beings, particularly in an inartificial state of society, the dog has ever proven himself the kindly defender of his life and property, as well as his faithful auxiliary in subduing other animals to his purpose. Without the assistance of the dog, man would not even yet, have obtained a beneficial dominion over the various races of wild animals of the earth, or have been able to watch, with sufficient care, those creatures formed for his food. If the proposition be true, and we take it for granted, it is: that originally the dog was of one species, or more strictly, of one variety, namely, the shepherd's dog; then we have only to fix in the mind the great variety of breeds that have sprung from this one, and to consider by what influence such changes have been brought about, to have abundant proof in support of the proposed theory. I think there are some sixty varieties or breeds from the original stock; including all the differences in size, color, shape, texture, and length of hair, from the silken-haired poodle—unnaturally nursed in the lap of some disappointed lady, as a substitute for something more desired and natural—up to the noble and sagacious friend of man, the Newfoundland dog. These bear respectively no stronger marks in their hereditary instinctive propensities, than in their anatomical construction, or adaptation to the performance of such duties as seem best fitted to their artificial natures. In confirmation of this, we have only to notice the peculiar and admirable adaptation of form and physical endowments in all these varieties. For instance, we see the water-spaniel has webbed feet to assist in swimming. The Newfoundland, a close, thick, long, and warm coat of hair, to protect him from the rigor of his arctic habitation. The graceful and fleet grayhound is the best possible model for fleetness—cutting the air like an arrow in his course. With one word more in relation to the dog, I will pass to another proof in support of my proposition. There is one kind of dog more used than any other for exterminating rats and mice in dwellings, storehouses, barns, &c. It was thought by some, that their ears and tails were in the way; consequently the one was cut off to within from one to two inches of the body, while the ears were shortened or trimmed, in a manner known to sportsmen as foxing. Very soon this was largely practiced on nearly all the dogs of this species, or, more properly speaking, this breed, until their *young were born with short or rounded ears and minus the caudal vertebræ.*

From the above facts, we see how remarkably prone is the animal economy to transmit the various propensities, and it is owing to the conformity of the body to these tendencies that we are indebted for all the pleasing and useful varieties of the canidæ. Let no human creature object to the analogous proof presented by comparison with the horse, or the dog; for the horse is certainly a noble and beautiful animal. The form and general qualifications of this creature are of a high order, and peculiarly susceptible of cultivation by art. The senses of smell and sight are remarkably acute, and the clearness of perception, excellence of memory, patient endurance, and gentleness, are not excelled in any other of the lower animals: while it may be said of the dog, that he is more faithful than even the most boasted among men. He is more constant in his affections, friendly without interest, and grateful for the slightest favors. Much more mindful of benefits received than injuries offered, he is not driven off by unkindness: he licks the hand that has just been raised to strike, and at last disarms resentment by submissive perseverance.

In order to extend the range of comparison for the purpose of showing how completely, nearly every order of animal creation is subject to the same natural laws of conformity, we will now descend low in the scale of being. We will select, for instance, the very lowest order of vertebrata —the fish—and while we crave indulgence for the length to which we have already carried this evidence by analogy, we trust we shall be able, at least in this one instance, to compensate for our tediousness by the novelty of the induction.

It has been the privilege of the writer to pay frequent visits to what is known as the Mammoth Cave, in the State of Kentucky. There is flowing through a portion of this cavern, a large, clear, and beautiful stream of water, in which is found a large number of fish, of the perch species.* Now, it will be understood, that the visitor can only explore this dark and dismal place by the aid of an experienced guide, who will bear a flambeau in his hand, giving sufficient light to enable one to view the remarkable wonders of this grotto. So actually does the economy in development conform to external or surrounding circumstances, that these fish are found to exist in this cavern stream without eyes. Indeed! of what earthly utility could eyes be to them, so long as they inhabit an abode, where the darkness is palpable?

As strange as this may seem, it is equally wonderful that these fish, taken from this gloomy darkness, and carefully placed where they are alternately exposed to light and darkness, will, in the short space of eleven years, propagate a race with the organs of vision perfect in development and function. Thus we have produced animals from the highest grade of physical perfection, in connection with the lowest order of vertebratæ, whose blood is but slightly above the

* There are several other kinds of fish inhabiting this cave, as well as large numbers of the crustacea without eyes.

temperature of the element they inhabit; and have endeavored to show how strikingly the animal economy is subject to the controlling influence of circumstances, and that, not only the shape and development of particular parts may be affected, but that the very organs themselves—not essential to vital existence—may be wholly dispensed with, or reproduced by the power and force alone of habits; or external circumstances. Having then, as we hope, made our object understood, we will at once proceed to the main purpose in our proposition.

It is true that man occupies the highest position in the scale of human creation, but this great superiority consists principally in his moral, and not in his physical, endowments. In his organic, or physical nature, he is subject to, and governed by, the same laws that govern and control the brute creation. Taking this for granted, we claim that just in proportion as the human mind is more active and powerful than that of the brute, so is his physical organization more likely to be influenced by mental excitement, or impressions of the mind. But this is not the point now for which we contend; we wish to establish this one position, that parents, who are defective in any part of their physical organization, are likely to transmit such defects to their progeny, and particularly diseases and defects of the teeth. I deem it a truth, that more than one-half of the children are born into the world with congenital, or natural, defects of the teeth.

This fact is so visible, that we even hear children say, "our whole family have bad teeth," or, as I have frequently heard people say, "it runs in our family to have bad teeth." Only a short time since I was amused at the remark of a little Miss, not twelve years of age, who said, "I take after my Ma in my teeth, and my Pa in my hair and eyes."

Having had my attention so frequently called to the diseased condition of children's teeth, and being often called upon to explain the primary cause of such decay, I have given this matter some little attention.

In thirteen cases of females, who are wearing entire upper sets, and nine out of the thirteen both upper and lower sets, complete. I am enabled, after a careful and thorough investigation, to give the following report: The oldest is sixty-seven years of age. The next five, between fifty-four and sixty. Four from forty-eight to fifty-three. Two from forty-five to forty-seven. The youngest forty-one.

The oldest has had nine children that lived to acquire all of the permanent teeth. The next, eleven; the third, thirteen; the fourth, eight; the fifth has ten; the sixth, seven; the ninth, eight; tenth, seven; eleventh, four; twelfth, seven; and thirteenth, five. With the exception of the oldest of these ladies, they have all borne children, under every variety of change, from sound and healthy teeth, throughout all the various stages of caries and other diseases usually witnessed, until, as before stated, four are wearing entire upper, and nine both upper and lower complete sets of artificial dentures. The eldest lady did not wear a plate till after her youngest child was born, though for six years before she had only two teeth left, but they were quite sound. Twenty-seven of these persons were born before their mother's teeth were much decayed. Seventeen were born when their mother's teeth were decaying and in an irritable condition. Twenty-nine were badly decayed, and the gums in some cases much diseased. Fifteen when the condition of the mouth was more healthy, in consequence of the removal of the diseased teeth, and as a matter of course, the general health and bodily vigor comparatively restored. While the remaining seventeen were born after a still more healthy condition was more permanently established, and they were all wearing plate, as before stated. Of the whole number of persons born of these thirteen mothers mentioned, seventy-four are personally known to the writer, and in relation to the other thirty-one, I have been able to secure from the immediate members of their respective families, all necessary and reliable information, by which he is enabled to give a minute description of the natural condition or qualities of all the teeth of one hundred and five persons, being the children of the thirteen mothers referred to.

Believing, however, the present purpose to demand only a general description, we shall not enter as fully into detail as may be thought proper to do on some future occasion. I could not give a better history of these cases than to say, that in very nearly every instance, when the children's teeth resembled those of the mother, and in some instances where the resemblance was only partial, they corresponded to the condition of the mothers' teeth during the respective periods of conception, gestation and lactation. Or in other words, those children born while their mother's teeth were sound, and particularly where both parents had good sound dentures, had teeth that would be classed healthy, sound, and of best quality. Children born at a later period, when the mother's teeth were beginning to decay, and causing some pain and derangement of the nervous system, with a partially deranged condition of the gums and mouth, showed also a correspondingly defective condition. Other children born at a still later period, (when the mother's teeth were badly decayed, with many necrosed and decaying portions of fangs, the alveolas exfoliating or undergoing absorption, with an excretion of sanies and pus into the cavity of the mouth, the gums highly diseased, inflamed and spongy, with fungoid growths springing up and discharging their fetid secretions, and the functionally deranged salivary glands pouring out an undue quantity of disordered fluid, during mastication, to be swallowed into the stomach, there to spend its virulence in completing its work of destruction,) only showed what those who have carefully noticed, may expect to find a true reflection of this sad picture.

It is with pleasure that I now call attention to a brighter and more pleasant phase of this case; and will state that where, by the kindly aid of nature, (who by the way may be often a good surgeon, but a poor dentist,) or the well directed assistance of art, the remaining diseased teeth and fangs have been removed, and the mouth has become healthy. I say it is with pleasure that under this condition of the mouth I am able to observe a transmission of health and vigor of body, but more especially a marked and decided improvement of the last children's teeth. Though in truth I must say, that even in this last instance, I was unable to discover so good a color and such freedom from nervous irritability or so good a structure of teeth, as in the case of the children born of the same mothers before their teeth had been injured by disease. Though not entering into the original purpose of this paper, I will state as a fact, that the general health of these persons did not seem to suffer to the same extent as the teeth would indicate; yet it was evident that those born while their mothers were laboring under general derangement of the mouth, were not so healthy, had not such physical capability of endurance. Their nervous systems were more easily deranged ; their stomachs did not digest food so readily, their hair become grey, or fell off much sooner; their skins were not so healthy and beautiful; their minds not so vigorous, and much less susceptible of cultivation. They suffered more in first dentition, and the temporary teeth were of a bad quality, and in most cases gave a great amount of pain and suffering.

In connection with what has been submitted, I beg leave to add the following, which I hope in a practical sense may not prove uninteresting. Within the last five years, with an ardent desire to secure as many facts as possible touching this point, namely, the congenital transmission of diseases of the teeth, I have, with no small amount of trouble, become enabled to make the following statement. Out of three hundred and eighteen cases of females from the age of twenty-four to thirty-five years, whose teeth were naturally bad, who have borne from three to nine children each, I have made the following observations. Two hundred and fifty-nine of the husbands of these ladies had naturally good eeth.

| | | | | | |
|---|---|---|---|---|---|
| Forty-two of the children | were | | fifteen years old. | | |
| Thirty nine | " | " | fourteen | " | " |
| Sixty-five | " | " | thirteen | " | " |
| Thirty-nine | " | " | twelve | " | " |
| One hundred and fifty-nine | | " | ten | " | " |
| One hundred and two | | " | eight | " | " |

One hundred and forty-eight over six and under eight years of age.

The whole number of children under six years was three hundred and two, not including those under the age of eighteen months. It will be readily seen that the above statement includes as before stated three hundred and eighteen mothers, all having teeth naturally bad, and the children of these mothers, eight hundred and eighty-one in number, from the age of eighteen months to fifteen years. Of these children, as before remarked, seven-tenths had teeth resembling, in very nearly all respects, those of their mothers, while the others resembled those of the fathers. I have been thus particular in stating the number of children at the different ages, as may be readily supposed, for the purpose of showing that an opportunity was had to observe not only the deciduous teeth, (or learn their entire history from reliable sources,) but in a majority of these cases to examine carefully the permanent teeth, excepting the dentes sapientiæ, to which little or no importance can be attached, at least so far as our present object extends. It will be seen in the instances already given, that a very large number of the children's teeth resembled those of their mothers, and in order to more fully test this matter, I have somewhat extended these observations merely for the purpose of comparing the relative tendency to transmit diseases of the teeth between the male and female, or in other words to learn, if practicable, whether the defects of the teeth were more likely to be inherited from the mother than the father. (It will be remembered in the two former cases the mothers'

teeth were defective, while the fathers' were not, or at least not to a very great degree.) I will, however, say, that these cases were selected fairly with reference to the one object, as above stated. I have been enabled to select only seventy-eight cases of gentlemen whose wives had teeth that were of as good an average quality as those of the gentlemen were, in the three hundred and eighteen cases previously stated, which I deemed essential in properly testing this matter. The ages of these gentlemen were from twenty-six to thirty-nine years. Their children numbered in all, (of suitable age,) two hundred and eighty-four. Of which number two hundred and nine had teeth more closely in all respects resembling those of their mothers, save in one important particular, which would only, at their youthful period of life, be detected by a practical dentist, and consisted simply in their being of an inferior quality, compared with the sound and superior teeth of many or most of their mothers. Before giving this subject any especial attention, I was prepared to find in such children as particularly resembled one parent more than the other, a corresponding similarity in the teeth. Experience has, however, taught me otherwise, for I have frequently noticed a remarkably close resemblance in the teeth, when scarcely a trace of likeness existed in other respects. Unfortunately, this deviation of the teeth from the general physiological tendency, is not in a majority of cases of the most desirable nature, even though one of the parents may have very good teeth; yet if the other has remarkably bad ones, and more especially where this defect depends on some constitutional depravity, will this imperfection be manifested in the teeth of nearly all the offspring. I am free to confess my surprise at this, for I had expected to find in the teeth of such children as resembled particularly one parent and not the other, not only a marked similarity of shape, size, and color of the teeth, but also a corresponding quality of texture and vital organism, or capability to resist all causes of disease.

A precisely similar instance may be given in children born of parents, one of whom may be of sound and robust constitution, while the other is hastening to the grave, borne down by the ravages of phthisis pulmonalis. One child, for instance, may so closely resemble the healthy parent, as to be readily recognized, by the school-day's acquaintance, as the child of the unhealthy father or mother, as the case may be, with no other clue to this fact than a very striking resemblance. Yet a few years may pass on, and as adult age begins to take the place of childhood, or youth the brilliant death-lighted eye, the hectic flush of cheek, with the constant evidence of irritability and disease of the lungs, the prophetic and ever-dreaded cough of the poor unfortunate consumption, tell in language too plain to be misinterpreted, that this early doom was the natural inheritance of this unfortunate child, from a lamented parent to whom no other trace of resemblance was ever shown.

Unfortunately in these sad cases there is no remedy, and but one prophylaxis, which may be given in few words. Hereditary phthisis pulmonalis will very generally manifest itself before the unfortunate subject of it is of marriageable age. Then, in the name of humanity, let reason prevail, and in the short space of a few brief generations, such a disease will scarcely be known among the human family. I can scarcely refrain from adding an expression of my full conviction of the absolute folly of giving any advice upon this important subject; for, though strange as it may seem, with regard to the two subjects which most of all others demand our earnest and deliberate reflection, does the mass of the world act with the most persistent folly—one is matrimony, the other is death. I am glad, however, that for the transmission of diseases of the teeth, a more practical remedy can be pointed out to all, and at the present day, is available to every class of society; therefore the responsibility of neglect in this very important matter, must justly attach itself to those who are wilfully culpable.

It may naturally be asked, What is the remedy proposed? As my object is to be as practical as possible, I

will at once answer this question. Remove from the mouth every cause of irritation and disease, which can be done only by first extracting every tooth and fang that cannot be well preserved. In the second place, have each cavity or decayed portion thoroughly and properly cleansed and removed, and where suitably prepared, filled with gold. in the best possible manner. Cleanse the teeth from all accumulations of tartar, or other extraneous substances, using the utmost care constantly to keep the teeth clean and healthy, which can be done only by such constant care and so much labor, that few, who have never experienced the real and absolute benefit resulting from it, could at first be induced to undertake. Let not only adults and parents pursue this course, but let them so educate their children, and guardians their wards, and in a very short time, indeed, will there be no longer such general and wide-spread destruction witnessed among the teeth of children and youth.

I am aware that there is much difficulty in the way of the mass of the people's ever comprehending the theory of congenital transmission of diseases of the teeth. In fact, it is difficult for the most learned and experienced to give a conclusive explanation or rationale of this interesting and wonderful fact.

It is not my purpose, in this paper, to attempt such a demonstration. I only purpose giving facts, believing such evidence to be more available with the majority of mankind than mere abstract theories, however plausibly they may be stated. I am also well convinced, that a popular belief has obtained that children's teeth are not influenced, to a very great degree, by the condition of the parents' teeth; and it is for the purpose of refuting this error, that I have introduced these statements.

This is no new theory, but is supported by many of the leading members of the profession. Prof. Harris tells us in a lecture on this subject before the class, (whose words I will quote as nearly as my memory will allow,) "That there is a closer resemblance in children to their parents, in the peculiarities of the teeth, than is often to be met with in any other structure or part of the body." "Not only is this observed," he says, in shape, size, color and composition, but in particular local *defects.*

Prof. Baumes, from whose work I will translate a few lines, clearly advocates this doctrine. He says, "The mammary affections of mothers also exert a strong influence on the milk teeth of their children, which are apt to have a hasty enamel, and a disposition to become impaired or decayed very early; and likewise, as a consequence, those of replacement are apt to be deprived of their enamel towards the edge, or but little covered with it, and granulated at the extremity, which leaves their surface depressed and the border more or less yellowish. When the father and mother are addicted to the drinking of wine or cider, the teeth of the children are of a grayish and dry enamel, marked with cracks lengthwise, and often covered with a black scum or exhalation near the gum—the latter is quite commonly—soft, inflamed and red. The scum of which I have spoken, is more difficult to remove with an instrument, than the most tenacious tartar."

I am aware that Prof. Baumes does not refer especially to congenital diseases, in his statement with regard to what he terms "The mammary affections of mothers," and I have only quoted this part of it, for the purpose of hinting at the cause, or one of the causes, of children's teeth, resembling in quality and composition those of the mother, more frequently than the father. The most interesting case I have ever seen, is that of a family now residing in the state of Kentucky, a description of which I noted in a memorandum book some four years ago, and will now copy.

The father, a son and two daughters, had respectively a protuberance on the anterior surface of the superior right canine, of an oblong spheroidal shape, with its longest diameter vertical to the tooth, extending nearly three-fourths of the length of the body or crown, and about twothirds of the width, presenting a greater convexity than the buccal surface of the tooth itself. What was more remarkable, was the peculiar kind of color or tint of this protuberance, which might be compared to that beautiful and delicate shade to be seen in the nearly white part of the the conch-shell. This family consisted of seven children, four having teeth as closely resembling those of the mother, as the other three did those of the father.

As I have before remarked, this is no new theory or discovery—but is a principle in physiology, connected with the history of our race, which should be more generally understood and practiced upon. The notice taken of this fact, or tendency in the parent, to transmit, congenitally, diseases of the teeth, was a matter of common remark, as long ago as when Jeremiah wrote. He speaks of it as a proverb, or common saying among the Jews. "The fathers have eaten sour grapes, and the children's teeth are set on edge." This, like all other proverbs in use, was based upon reality and fact, and that, too, of so general a character, as to be familiar to all, which rendered it at once a useful and interesting manner of illustration. It is also, at the present day, a matter of notoriety, that the children born of parents who live in the champagne districts of France, where they frequently eat sour grapes and drink an acid wine, have teeth of a very inferior quality. The enamel is of a blue tinge, very hard and brittle. Such teeth are exceedingly sensitive to the impressions of heat and cold, and give great pain and unpleasant feeling, whenever it becomes necessary for the dentist to use a file—an operation to which such patients submit with the greatest difficulty. It may be said that this injury sustained is owing to the fact, that the children themselves make use of the grapes and wines.

It is very properly urged against this explanation, that the children born of parents, who removed from this part of France during the period of gestation to other parts or countries where these deleterious agents could not have been procured by them, still exhibit, in the appearances of their teeth, the same defective condition, as before described. From observation, I am satisfied that the teeth, through their primary organism, derive far more permanent impressions, during fœtal formation, than almost any other part of the system. An impression, which no care or skill on the part of the physician or dentist, can subsequently wholly remove. For instance, the mother, while pregnant, and especially during the more advanced stages of gestation, may be attacked with morbilli, or scarlatina; the child may show both, in the deciduous and permanent teeth —symptoms of atrophy, or as Prof. Harris expresses it, odontatrophia. I am aware this disease, from the above cause, is of rare occurrence—and I only mention it to show the extent to which the organs, concerned in the formation of the teeth in all their parts, are liable to functional interruption, and that too, at a time as early even as the first development of fœtal existence.

Let us now, in closing, review briefly the ground which we have traversed. We have shown that the greater law, by which species are preserved, namely, that the characteristics of the parents shall descend to the progeny, so that, while individuals change, the type ever remains, embrace not only these qualities, which we generally call *natural*, but also, whatever accidental peculiarities may have been impressed with unusual force upon the constitution of the progenitors.

This fact we have illustrated by several conclusive examples, from different orders of the brute creation, and the consideration of these examples prepares us to find analagous transmissions in our own species. One most important class of which we have in especial view—the transmission of morbid peculiarities of the teeth. It is needless to repeat the statistics already given on this point; but from them results inevitably the conclusion, that the condition of the parents' teeth, especially the mother's, at the time when the offspring receives the vital impulse and direction on which its physical history must depend, will surely decide the quality and morbid predisposition of the same organs in the child, so that a mother may have children with excellent teeth, while her own are yet unimpaired, a second group with teeth that speedily fail; because they were formed while the mother's were undergoing destruction; and, finally, a third group, with teeth little inferior to the first, after her mouth has been relieved of all disease. These facts are strictly and unanswerably proven, and they point to their own deduction.

In no other instance are the morbid results of hereditary transmissions at once so sad and so entirely subject to human prevention; and, while it seems an Utopian dream of the physiologist, that parents will one day realize their infinite obligations to their unborn children, in untiring self-control, in bodily and spiritual health and purity, it is happily not too much to hope, that in this point a strong voice of conviction may reach every mother in the land, until it may be said of the mothers of new generations as of the Beloved of Solomon, "Thy teeth are like a flock of sheep, that are even shorn, which come up from the washing, whereof every one bear twins, and none is barren amongst them."

